# Potential Impacts on Treated Water Quality of Recycling Dewatered Sludge Supernatant during Harmful Cyanobacterial Blooms

**DOI:** 10.3390/toxins13020099

**Published:** 2021-01-29

**Authors:** Kanarat Pinkanjananavee, Swee J. Teh, Tomofumi Kurobe, Chelsea H. Lam, Franklin Tran, Thomas M. Young

**Affiliations:** 1 Department of Civil & Environmental Engineering, University of California Davis, Davis, CA 95616, USA; kanpin@ucdavis.edu; 2 Department of Anatomy, Physiology, and Cell Biology, School of Veterinary Medicine, University of California Davis, Davis, CA 95616, USA; sjteh@ucdavis.edu (S.J.T.); tkurobe@ucdavis.edu (T.K.); chylam@ucdavis.edu (C.H.L.); fdtran@ucdavis.edu (F.T.)

**Keywords:** harmful cyanobacteria, cyanotoxins, conventional water treatment

## Abstract

Cyanobacterial blooms and the associated release of cyanotoxins pose problems for many conventional water treatment plants due to their limited removal by typical unit operations. In this study, a conventional water treatment process consisting of coagulation, flocculation, sedimentation, filtration, and sludge dewatering was assessed in lab-scale experiments to measure the removal of microcystin-LR and *Microcystis aeruginosa* cells using liquid chromatography with mass spectrometer (LC-MS) and a hemacytometer, respectively. The overall goal was to determine the effect of recycling cyanotoxin-laden dewatered sludge supernatant on treated water quality. The lab-scale experimental system was able to maintain the effluent water quality below relevant the United States Environmental Protection Agency (US EPA) and World Health Organisation (WHO) standards for every parameter analyzed at influent concentrations of *M. aeruginosa* above 10^6^ cells/mL. However, substantial increases of 0.171 NTU (Nephelometric Turbidity Unit), 7 × 10^4^ cells/L, and 0.26 µg/L in turbidity, cyanobacteria cell counts, and microcystin-LR concentration were observed at the time of dewatered supernatant injection. Microcystin-LR concentrations of 1.55 µg/L and 0.25 µg/L were still observed in the dewatering process over 24 and 48 h, respectively, after the initial addition of *M.* *aeruginosa* cells, suggesting the possibility that a single cyanobacterial bloom may affect the filtered water quality long after the bloom has dissipated when sludge supernatant recycling is practiced.

## 1. Introduction

An increase in the frequency and severity of harmful cyanobacterial blooms has been observed worldwide [1,2,3,4,5,6]. Cyanobacteria are prokaryotes, but have been widely referred to as “blue-green algae” because of their ability to perform photosynthesis and similarity in size and color to many algal species. Multiple cyanobacteria species grow in a typical bloom, but the most abundant and common cyanobacteria found in harmful algal blooms across the globe is *M. aeruginosa* [7,8,9]. There are many important factors controlling the growth *of M. aeruginosa* in an aquatic environment including physical disturbance, light, temperature, nutrients, and grazing. Eutrophication linked to the increase of nutrients including nitrogen (N) and phosphorus (P) in water systems is generally considered as one of the major factors that favor the development of planktonic cyanobacterial blooms [10,11]

A critical problem caused by *M. aeruginosa* is associated with their potential to release cyanotoxins [11,12,13,14]. These toxins have the ability to cause human health problems such as liver cancer or neurotoxic effects [15]. The most common and intensively identified toxin class is microcystins (MCs), especially the microcystin-LR (MC-LR) form, in which L and R stand for the distinguishing amino acids leucine and arginine, respectively [12,13,14]. Due to the toxicity of MCs, the WHO has established a drinking water guideline of 1.0 µg/L of MC-LR equivalents [16]. The United States has not established a national MCs drinking water standard; consequently, MCs standards vary by state.

To protect people from MCs exposure, physical and chemical treatment methods are usually applied to remove algal cells and associated toxins during water treatment. Coagulation, flocculation, and sedimentation is a commonly used treatment sequence during drinking water treatment, supplying many benefits such as ease of operation, low cost, rapid reaction rates, and high efficiency [4]. These preliminary treatments have been shown to achieve 78% to 92% removal of cyanobacteria cells [17,18]. Metal salt coagulants such as alum (Al(SO_4_)_3_·18H_2_O) are effective because they can neutralize the negative surface charges present on cyanobacterial cells at natural water pH levels [19]. Charge neutralization promotes floc formation, allowing cyanobacteria to settle out during subsequent sedimentation.

Although the conventional treatment method of coagulation-flocculation and sedimentation is considered effective in removing cyanobacteria cells [4,12,20], the process only neutralizes the charges on the surface of the cells but does not deactivate cellular functions [21,22,23]. Thus, it is possible that the cells that were not removed by the sedimentation process could repopulate in downstream filtration media and negatively impact treated water quality [24]. A rapid increase of cyanobacteria cell counts caused by incomplete deactivation of cyanobacteria in a water treatment plant has been reported by Gad and El-Tawel, (2016) [23].

Cyanobacterial regrowth in the filter media may not be the only mechanism for the persistence of, and a possible increase in, the cyanobacterial populations in water treatment plants. Many water treatment plants recycle settling basin supernatant to the plant headworks, potentially allowing cyanobacterial regrowth and cyanotoxin redistribution [25,26,27]. The accumulation and subsequent regrowth of cyanobacteria and the excretion of cyanotoxins in sludge from the dewatering process may lead to prolonged effects of cyanobacterial blooms in drinking water treatment operations [26], particularly since cyanobacteria cell lysis may occur and result in the release of additional cyanotoxins.

**Figure 1:**
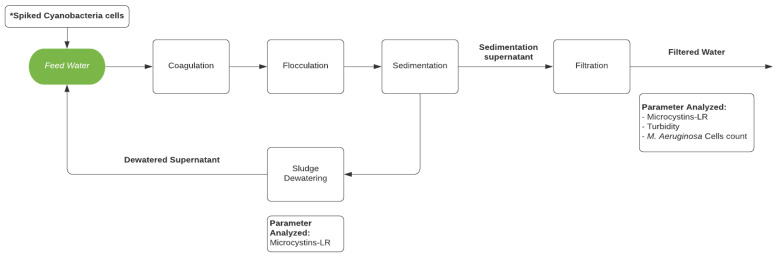
Schematic of the lab-scale water treatment system. * Only for experiments AE (A-Spiked) and BE (B-Spiked).

Therefore, the objectives of this study were to: (1) investigate the accumulation of cyanobacteria cells and microcystins in conventional filter media during operation of a lab-scale drinking water treatment system experiencing a simulated bloom of toxigenic *M. aeruginosa* cells, (2) explore the possibility of cyanobacteria repopulation and microcystin excretion during the dewatering process, and (3) examine the effect of dewatered supernatant recycling on the quality of filtered water. The lab-scale treatment system, depicted in Figure 1, includes a conventional process train of coagulation, flocculation, sedimentation, and filtration.

## 2. Results

### 2.1. Feed Sample Characteristics

Source water obtained from the intake to the Woodland and Davis Clean Water Association (WDCWA) treatment plant on two collection dates featured relatively low turbidity (<32 NTU), with a pH of 7.6 and 8.2 for experiment A and experiment B, respectively, and no detectable concentrations of MC-LR (Table 1). Experiments A and B are two pilot-scale experiments conducted under the same operating parameters at different times to ensure the reproducibility of the results.

The lab-scale treatment system used in these experiments (Figure 1) is a batch system designed to mimic the behavior of a full-scale treatment process that includes coagulation, filtration, sludge dewatering, and supernatant recycle to the filters. Feed water supplied to the system consisted of WDCWA raw water with (spiked experiment, E) or without (control experiment, C) the addition of laboratory cultured *M. aeruginosa* cells. The water quality of the spiking solutions and the spiked feed water for each experiment are reported in Table 2. The turbidity of the feed water is considered to be the same as for the raw water since the change in turbidity was insignificant (<0.1 NTU). The *M. aeruginosa* cell counts in the experimental water samples are controlled to be 1 × 10^6^–1 × 10^7^ cells/mL, which is within the range of *M. aeruginosa* cell counts commonly observed during bloom events [4,18]. Also shown in Table 2 are the flow rates, dewatered sludge supernatant injection times, and optimal coagulant dosages applied (as determined by separate jar tests) for each experiment.

**Figure 2:**
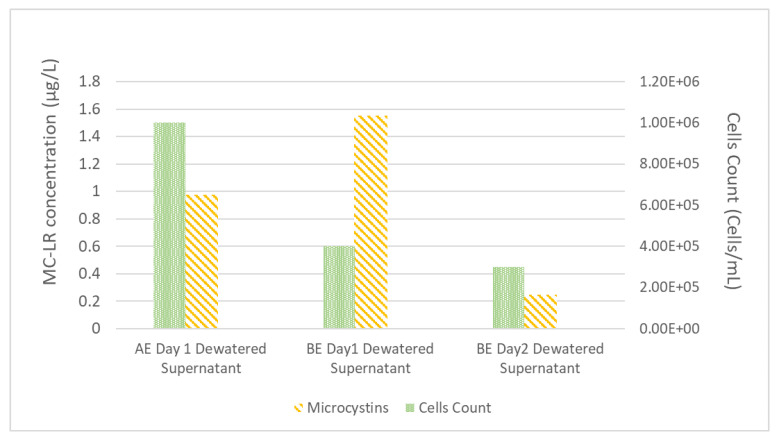
Experiment A (AE) and B (BE) dewatered supernatant composition.

After the process of coagulation-flocculation and sedimentation was completed, the supernatant of the settled sample was transferred into the filtration process and the settled sludge was concentrated in the dewatering system. After 24 h of dewatering process, the dewatered process supernatant (dewatered supernatant) was removed and mixed with WDCWA raw water to serve as experimental feed water during the second day of the experiment; this simulates the process of recycling liquids from dewatering operations to the feed water as practiced at many treatment facilities. MC-LR concentrations and *M. aeruginosa* cell counts for dewatered supernatant for experiments AE (A-Spiked) and BE (B-Spiked) are shown in Figure 2.

Data shown in Figure 2 follow the same trend as the data shown in Table 2 with higher MC-LR and lower cell counts for experiment B than experiment A. The MC-LR concentrations of dewatered supernatant for experiments AE and BE (Figure 2) are higher than the feed water MC-LR concentrations (Table 2), this result highlights the potential for *M. aeruginosa* cells to be lysed during the coagulation and flocculation process and also during the dewatering process.

### 2.2. Filtration Performance

**Figure 3:**
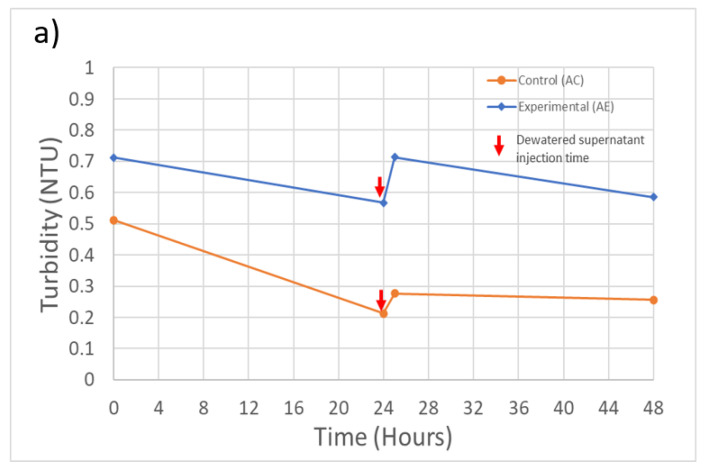

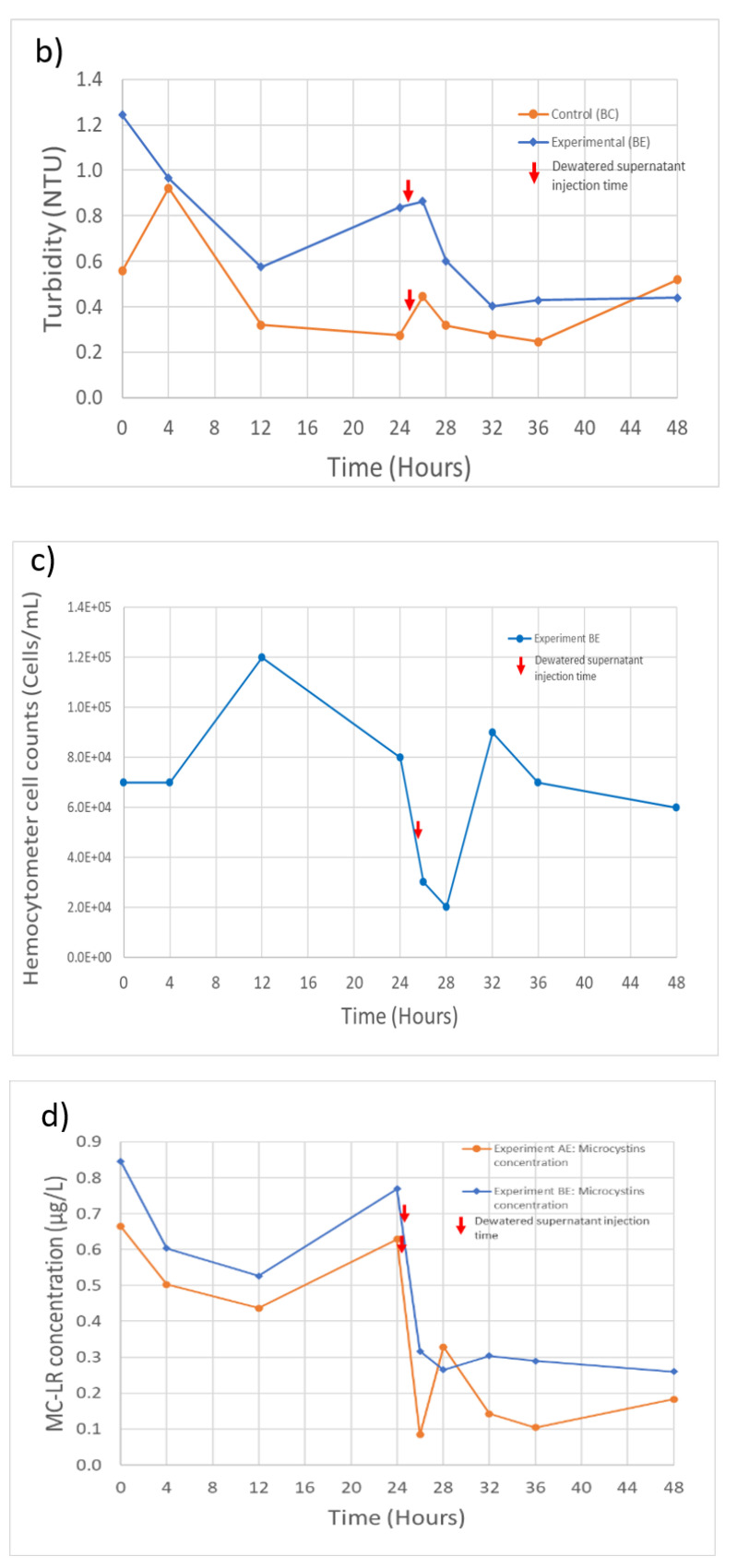
Experiment A and B filtration process effluent quality. (**a**) Experiment A turbidity (*n* = 3), initial concentration: 31.7 NTU; (**b**) Experiment B turbidity (*n* = 3), initial concentration: 13.2 NTU; (**c**) Experiment B *M. aeruginosa* cell counts (*n* = 1), initial concentration: 1.6 × 10^6^ cells/Land; (**d**) Experiment A and B MC-LR concentrations (*n* = 1), initial concentration: 0.29 and 0.53 µg/L, respectively. Error bars represent standard deviation from measurement replicates.

Overall, during these experiments, including following the injection of dewatered supernatant, the filtration system maintained an effluent turbidity level below the 1 NTU limit required under U.S. EPA regulations (Figure 3). The only exception was at the initial time point for experiment B, which slightly exceeded the 1 NTU limit. During experiment A, the system removed 99.0% of the initial 31.8 NTU (Table 2) turbidity for the control feed solution and 98.0% for the spiked feed solution (Figure 3a). During experiment B, the filtration system removed 96.7% of the initial 13.2 NTU turbidity from the control feed solution and 94.7% from the spiked feed solution (Figure 3b). During both experiments, the recycling of dewatered supernatant produced a sudden increase in the effluent turbidity, but the increase was not sufficient to cause the system to exceed the 1 NTU effluent guideline.

*M. aeruginosa* cell counts for the filtration process effluent in experiment B are shown in Figure 3c (effluent cell counts are not available for experiment A). The treatment process in experiment B was able to remove 95.8% of the initial 1.6 × 10^6^ cells/mL (Table 2). An increase in the cell counts following injection of dewatered supernatant can also be observed, but the timing of the increase is delayed in comparison with the time of the turbidity increase. The cause of this difference in timing is not clear, but it could mean that the prior turbidity increase may not be solely due to the influence of the injected *M. aeruginosa* cells.

MC-LR concentrations in filter effluents are shown in Figure 3d. The treatment process removed 55.6% and 38.5% of the MC-LR present in the feed solutions during experiments A and B, respectively. However, it can also be seen in Figure 3d that, at some time steps, the MC-LR concentration exceeded the concentration of MC-LR in the feed solution (Table 2). The additional MC-LR may have been released by cell lysis during the coagulation and flocculation steps, releasing the intracellular MC-LR into the dissolved phase, thereby increasing the extracellular MC-LR that was being measured. A further increase in the MC-LR filter effluent concentrations during both experiments can also be seen after the injection of the dewatered supernatant at the 28- and 32-h time points for experiments AE and BE, respectively. Although the MC-LR concentrations did not exceed the WHO guideline of 1 µg/L at any point in time during these experiments, the result reinforces previous findings that conventional water treatment systems are not highly efficient in removing extracellular MCs [28].

### 2.3. MC Retention in Filter Media

It is challenging to determine the number of *M. aeruginosa* cells retained within the filter media because of the large amount of other suspended solids also retained. Consequently, only the total MC-LR concentration within the sand media was investigated. After a full cycle of filter operation, the sand media had retained a total MC-LR concentration (extracellular and intracellular) of 14.89 µg/L (1.04 µg/L extracellular MC-LR and 13.85 µg/L calculated intracellular MC-LR). To put this value in context, this represents approximately 29.5% of the total amount of MC-LR (Table 2) delivered to the filter during experiment BE. This data supports a conceptual model in which intracellular MC-LR from the *M. aeruginosa* cells retained is the primary component of the total MC-LR concentration within the sand filter; this is consistent with the relatively low removal efficiency for extracellular MC-LR and the simultaneous high level of removal of cyanobacteria cells by these sand filters.

### 2.4. Effects of Dewatered Supernatant Recycling

**Figure 4:**
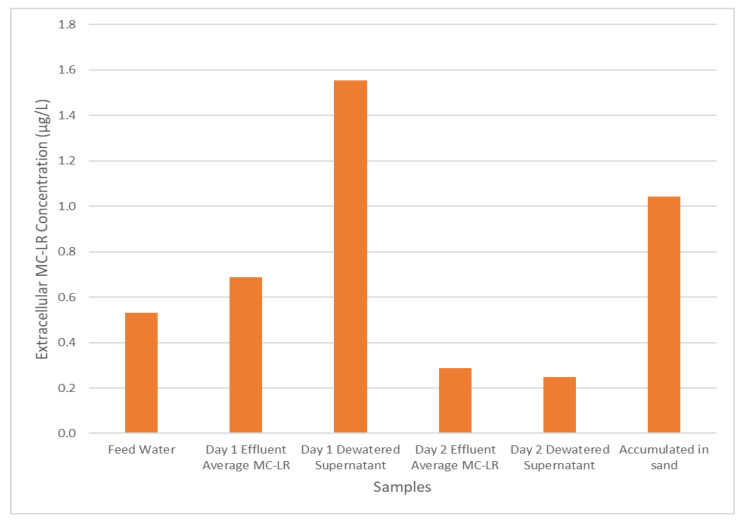
Experiment BE, extracellular MC-LR concentration throughout the water treatment process.

A key goal of these experiments was to determine whether the recycling of dewatered supernatant could serve to prolong the detrimental effects of cyanobacterial bloom events on treated water quality. The concentration of extracellular MC-LR across the treatment system during experiment B is shown in Figure 4. After the feed water enters the pilot system and undergoes coagulation, flocculation, and sedimentation, the MC-LR concentration increases, presumably due to the rupture of cells caused by the mixing processes; this effect is also reflected in the dewatered supernatant MC-LR concentration shown in Figure 4.

The MC-LR originally retained primarily in an intracellular form are now able to move through the process and enter the filtration process. However, as mentioned previously, the filtration process exhibits relatively low removal of extracellular MC-LR, resulting in its release in the filtrate solution. Further, examining the MC-LR concentration in day 2 dewatered supernatant at the end of the operation, it can be seen that MC-LR remains in the supernatant waiting to be recycled back into the system during the next operation period along with the MC-LR accumulated in the sand media for the previous 48 h of operation time. The retained MC-LR could be released from the filter media during the backwashing operations. Thus, even for a cyanobacterial bloom that affected feed water for only 24 h, the effects of the toxins and cyanobacteria from the bloom may persist much longer than one treatment system residence time if the plant recycles dewatered supernatant and/or the filter media backwash water.

**Figure 5:**
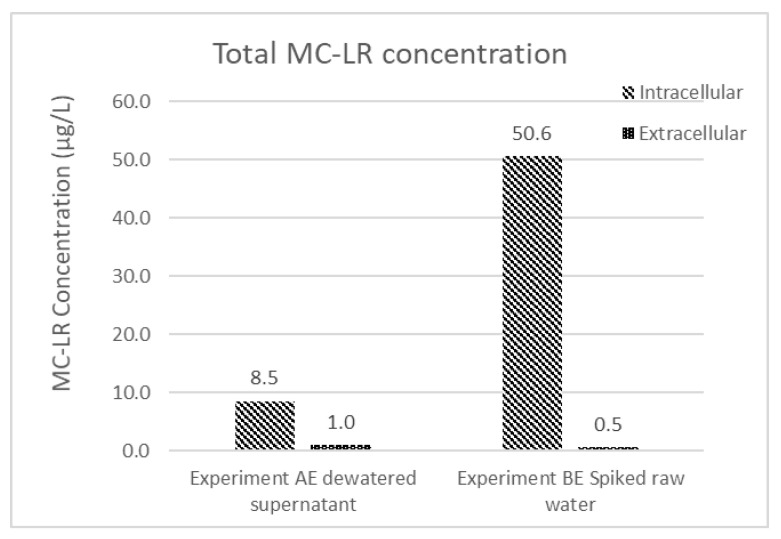
Extracellular and intracellular MC-LR concentration of experiment AE dewatered supernatant and experiment BE spiked raw water.

Even though the MC-LR concentration in the effluent water is below the WHO guideline, the measured concentration only considers the extracellular fraction of the water’s total MC-LR content. To consider the potential amount of MC-LR that could be contributed to the solution if the intracellular portion was released to the solution, intracellular and extracellular MC-LR concentrations were measured for the dewatered supernatant and spiked feed water from experiments AE and BE, respectively (Figure 5). Most of the total MC-LR concentration (90%–98%) in these two samples is intracellular. Although MC-LR within intact cells can be efficiently removed by conventional treatment operations, the presence of high fractions of MC-LR in retained solids makes the permanence of this removal dependent on subsequent solids handling decisions.

## 3. Discussion

### 3.1. The Effectiveness of the Conventional Treatment Process

The results from the water quality analysis demonstrate the capability of the pilot experimental system to remove turbidity, *M. aeruginosa* cells, and MC-LR to levels below the EPA and WHO regulations. Our finding that *M. aeruginosa* cells were removed with greater than 95% efficiency from the feed water by the processes of coagulation, flocculation, and sedimentation are similar to results reported by Chow et al. 1999 [29] but lower than found in a pilot-plant scale study by Zamyadi et al. 2013 [18]. Though the removal efficiency is high, the total number of *M. aeruginosa* cells remaining in the effluent stream might still cause problems in downstream unit operations, such as disinfection. It is worth noting that the microscopic observation method employed here using a hemacytometer is tedious and challenging. Implementation of a more precise cell counts method, such as via polymerase chain reaction (PCR) or flow cytometry is recommended.

Removal of the extracellular MC-LR cyanotoxin, however, was far less efficient, with overall removals below 50%. Although low, this is still higher than the results shown previously by Ho et al. 2006 [24]. The higher removal efficiency of intracellular, particle-associated cyanotoxins in comparison with MC-LR in its dissolved form is to be expected during conventional water treatment operations such as coagulation-flocculation and filtration, which are targeted at the removal of particulate matter. Other filter configurations may be more successful in removing MC-LR and related compounds; previous research indicates that the presence of biofilms on filter media, for example, can assist in degrading cyanotoxins; however, the bacterial biofilm required at least 4 days to form and be functional [13,24].

Regarding the extracellular MC-LR concentration, it can be seen that at the initial state of the treatment process (0 h) the MC-LR concentration in the filtered effluent is higher than the initial concentration in both experiments AE and BE. The increase in the MC-LR concentration observed here differs from findings reported previously by Chow et al. 1999 [29], who found no significant increase in MC-LR concentration following coagulation-flocculation. It is possible that the rapid mixing step used here caused additional cell lysis and contributed to the increased extracellular MC-LR in the effluent water.

### 3.2. The Effects of Dewatering Supernatant Recycling

Due to the high removal of *M. aeruginosa* during the coagulation-flocculation process, cyanobacteria cells accumulate in the sludge, which is subsequently sent to the dewatering system. The dewatering experiment revealed that intracellular MC-LR are released to the solution during this phase, exhibiting a 192% increase in extracellular MC-LR concentration compared with the concentration in the feed solution (Figure 4). If this extracellular MC-LR is recycled into the treatment system, it has the potential to extend the effects of a cyanobacterial bloom event on the water treatment system. The increased duration may be longer than the hydraulic residence time of the treatment system as both the dewatered supernatant from the 2nd-day operation and the MC-LR that may be released from the filter media through the backwashing process could be recycled back into the treatment system.

As mentioned previously, the treatment simulations performed in this study only addressed the effects of a cyanobacterial bloom on the coagulation-flocculation and filtration processes and do not address possible impacts on the disinfection process. If the disinfection process were included, a lower concentration of *M. aeruginosa* cells and microcystins might be achieved, as shown by previous studies [13,14,30,31,32] Even though the disinfection process may oxidize the cyanobacterial membrane and initiate the release of intracellular microcystins, this may produce other hazards in the form of increased concentrations of disinfection by-products including trihalomethanes (THM), haloacetic acids (HAA), and N-nitrosodimethylamine (NDMA) [14,29,33,34,35,36].

### 3.3. Future Extensions of the Research

Future extensions of this research would include upgrading the batch simulation approach used in these experiments to a continuous treatment process, which may result in a more accurate estimation of the MC-LR concentrations in effluents from conventional drinking water processes. The addition of a chlorination process to the simulated treatment train would allow the effects of recycling cyanobacteria-laden dewatered supernatant on the amount and product distribution of disinfection-by-products to be explored. Another worthwhile avenue for future research aimed at reducing cyanotoxin impacts is to explore possible origins of cyanobacterial blooms in various water sources; this could inform watershed management approaches that minimize cyanobacterial blooms to reduce the probability that cyanotoxins and/or elevated levels of disinfection-by-products will impact final drinking water quality.

Though a pilot-scale experiment might achieve the desired removal efficiency and better imitate the actual water treatment process, conducting a plant-scale experiment is ultimately recommended. Investigating these processes in a real water treatment plant will alleviate problems associated with limited water sample size and allow more robust analysis of the performance of each individual process. However, if a plant-scale experiment were conducted, the operators must ensure that the effluent from the experiment does not enter the drinking water distribution system, as it might be harmful for the community surrounding the plant.

## 4. Conclusions

Overall, the results of the current study have shown the possibility of an increase in turbidity, cell density, and MC-LR concentration caused by the recycling of dewatered supernatant and the possible extension of the water quality effects from seasonal cyanobacteria blooms. Thus, we strongly advise against the recycling of dewatered supernatant during cyanobacterial bloom periods, redirecting these materials instead to the sludge treatment process. These steps will help minimize the duration of cyanobacterial bloom impacts on finished water quality. However, a plant scale study to confirm these results in field-scale water treatment facilities is recommended.

## 5. Materials and Methods

### 5.1. Materials and Reagents

The microcystin-producing strain *M. aeruginosa* was obtained from the University of Texas Culture Collection of Algae (UTEX, https://utex.org/, UTEX Culture ID: LB2385, Austin, USA) and cultured in a synthetic cyanobacteria growth media, CB media (adapted from Shirai et al. 1989 [37]) under fluorescent lamps in an incubator with a controlled temperature of 25 °C. The cyanobacteria cells were harvested at 2 weeks of incubation during the logarithmic growth phase of the cyanobacteria and the initial cell counts were analyzed using Hausser Scientific Hemacytometer (Fisher Scientific, Pittsburgh, PA, USA). The MC-LR standard was purchased from Enzo Life Sciences (Farming Dale, NY, USA). The coagulant aluminum sulfate hydrate (Al_2_(SO_4_)_3_·18H_2_O) was purchased from Sigma-Aldrich (St. Louis, MI, USA) and the solutions were prepared with deionized (Milli-Q) water prior to the experiments; new solutions were prepared for each experiment. The water source chosen for the study was the Sacramento River; samples were collected twice from the WDCWA treatment plant raw water intake pipe on 13 February and 27 February 2020. The raw water samples were then stored in a refrigerator at 4 °C. Prior to starting experiments each day, the water samples were taken out and allowed to attain room temperature.

### 5.2. Experimental Setup

#### 5.2.1. Optimum Dosage Jar Test Experiment

In order to determine the optimum coagulant concentration for the lab-pilot scale experiment, a series of jar test experiments were conducted using a Programmable Jar tester (Phipps &Bird Model PB-900, Fisher Scientific, Pittsburgh, PA, USA) with a 2000 mL beaker at room temperature (23 ± 1 °C). The coagulant was added to 1000 mL of *M. aeruginosa* spiked feed water with a rapid mixing speed of 250 rpm for 60 s [17] After the coagulation process, the sample underwent slow mixing at 40 rpm for 30 min. Then, the samples were allowed to settle for 30 min. The supernatant from the jar test experiments were collected to determine the turbidity and *M. aeruginosa* cell counts.

#### 5.2.2. Filtration Column Preparation

The glass filtration column (length 20 cm, internal diameter 3 cm) was packed with an autoclaved sand filter media with a bed height of 15 cm. Milli-Q water was pumped through the bed in a downflow configuration until steady state was achieved at the designated flow rate. At that time, the Milli-Q water was replaced with the supernatant from the preliminary treatment process.5.2.3. Lab-Scale Experiments

The controlled sample without any spiking of *M. aeruginosa* cells (control, C) and the feed water with spiked *M. aeruginosa* to imitate cyanobacterial blooms entering the water treatment system influent (experiment, E) were prepared for both experiments A and B.

Stock *M. aeruginosa* cells suspensions with a density of 20 × 10^9^ cells/mL were prepared and resuspended in raw water to achieve a cell suspension of 1 × 10^6^ cells/mL for experiments AE and BE. No *M. aeruginosa* cells were added to the feed water for experiments AC and BC. After the resuspension of the *M. aeruginosa* stock solution, both experiments underwent the same procedures. The spiking of cyanobacteria cells into the raw water was only done on the first day of the experiment.

Jar test experiments were performed to represent the coagulation-flocculation and sedimentation processes during water treatment using the same configuration as the jar tests conducted to determine optimum coagulant dosages.

The supernatant from the jar test experiment was then fed to the filtration process. The filtration system was expected to be able to operate for 48 h without backwashing. A 1 L sample was collected from both experimental conditions (C and E) at 0, 4, 12, 24, 26, 28, 32, 36, and 48 h after filtration and stored in the refrigerator at 4 °C for a maximum of 48 h, before undergoing the microcystin analysis. These water samples were analyzed for turbidity and cyanobacteria cell counts immediately after being collected.

All the sludge from the jar test was collected, combined, and transferred to a separate dewatering container for each of the experiments and allowed to settle for 24 h. After 24 h of dewatering, the supernatant from the process was separated into two parts, 500 mL of the sample were used for MC-LR concentration analysis and the remainder was transferred to the second-day feed water to imitate the recycling of dewatered supernatant in a conventional water treatment system. The combined water was processed through the jar test experiment again to simulate the preliminary treatment.

### 5.3. Water Quality Analysis

#### 5.3.1. Turbidity

The water effluent samples from each time step were analyzed for turbidity using a HACH 2100AN turbidity meter (HACH, Loveland, CO, USA). The instrument was calibrated according to the manufacture’s specifications for every 24-h time step.

#### 5.3.2. Analysis of Microcystins

In the absence of suitable standards for most microcystin variants, the microcystin concentration in this experiment for all samples was expressed as equivalent of microcystin-LR (MC-LR) [38,39,40].

Prior to the liquid chromatography MC-LR analysis, the samples were concentrated using solid-phase extraction cartridges (Waters, Oasis SPE, Milford, CT, USA). The MC-LR concentration was analyzed by using a Liquid Chromatography-Quadrupole Time-of-Flight-Mass Spectrometer (Agilent 1260 Infinity HPLC coupled with an Agilent 6530 QTOF-MS, LC-QTOF-MS, Santa Clara, USA). Sample volumes of 10 µL were injected into the column (Agilent Zorbax Eclipse C18, Santa Clara, CA, USA) at a flow rate of 0.35 mL/min. Negative Electrospray Ionization (-ESI) mode was selected for the analysis. The concentration of the MC-LR was determined by the calibration of the peak areas.

#### 5.3.3. Analyzing the Sand Filter Media after the Experiment

After the breakthrough point for either the turbidity or water height was reached in both control and experimental filter beds, the filter media was removed from the glass column and transferred into separate Erlenmeyer flasks. Deionized water (Milli-Q, 500 mL) was then added to the flasks, and flasks were mechanically shaken for 24 h to remove deposited cyanobacteria cells and MC-LR from the media surface. Then, the supernatant from the process was filtered through a glass filter module. The filter paper was collected and cut into 1 mm × 1 mm pieces and sonicated in 20 mL of Milli-Q water for 2 h, after the completion of the sonication the sample was centrifuged at 3500 rpm for 5 min. Both the filtered and the centrifuged samples were combined and analyzed for total MC-LR concentration.

## Figures and Tables

**Table 1:** Raw water characteristics at the WDCWA treatment plant intake.

Experiment	Collection Date	Turbidity	Cell Counts	MC-LR	pH
		NTU	Cells/mL	µg/L	
A	13 February 2020	31.8	<1 × 10^4^	<LOD *	7.6
B	27 February 2020	13.2	<1 × 10^4^	<LOD *	8.2

* Limit of detection (LOD): 0.005 µg/L.

**Table 2:** Feed water and spiking solution characteristics and Lab-scale experimental conditions.

Experiment		Cyanobacteria Solution		Cell Count	MC-LR		Feed Water Average Flow Rate	Time of Injection of Dewatered Sludge Supernatant
	Optimal Coagulant Dosage				Extracellular	Total		
	mg/L	Cells/mL	(MC-LR) µg/L	Cells/mL	µg/L		mL/s	Hours
A-Control (AC)	30	-	-	-	<LOD *	-	13	24
A-Spiked (AE)	30	2.00 × 10^8^	11.41	1.0×10^7^	0.29	-	13	24
B-Control (BC)	30	-	-	-	<LOD *	-	14	25
B-Spiked (BE)	30	6.50 × 10^7^	10.17	1.6×10^6^	0.53	50.57	14	25

* Limit of detection (LOD): 0.005 µg/L.

## Data Availability

Data available upon request.
